# Estimation of the committed radiation dose resulting from gamma radionuclides ingested with food

**DOI:** 10.1007/s10967-014-2926-3

**Published:** 2014-01-23

**Authors:** Piotr Godyń, Agnieszka Dołhańczuk-Śródka, Zbigniew Ziembik, Ewa Moliszewska

**Affiliations:** Opole University, ul. kard. B. Kominka 6, 45-035 Opole, Poland

**Keywords:** ^ 137^Cs, ^ 40^K, Food, Effective weighted dose

## Abstract

The objective of the study was to estimate the value of the radiation dose absorbed in consequence of consumption of popular food products for individual age groups. Potatoes, corn and sugar beet were selected for the study. Edible parts of these plants were collected in experimental fields of the KWS Lochów Polska Sp. z o.o. seeding company in Kondratowice (Poland). On the basis of the obtained study results, it can be stated that in consequence of consumption of the selected food products, people may receive increased doses from both natural and artificial radioactive isotopes. The doses calculated for several age groups do not show any health hazards in consequence of consumption of the tested food. One of the determined radionuclides was^ 137^Cs; however, its presence in the absorbed dose is lower than the doses from natural radioactive isotopes, in particular^ 40^K.

## Introduction

Natural background radiation originates mainly from uranium–radium, uranium–actinium, thorium series elements and radionuclide ^40^K, as well as from nuclear transformations of such gases as nitrogen, oxygen or argon under the influence of highly energetic cosmic radiation [1, 2].

Activity concentration of natural radioactive isotopes in a given area is usually stable and low, although it may vary in consequence of human activity. The increase of radioactive substances content in environment can be caused by mining industry, firing fossil fuels, use of phosphorus fertilizers and discharging mining waters to water reservoirs. Also, artificial isotopes may be found in natural environment when released in consequence of, for example, various breakdowns, careless operation of industrial facilities, nuclear weapon tests and nuclear accidents such as one that happened in Chernobyl in 1986. The most important natural radiation sources are ^ 40^K and the elements from the series  ^235^U and^ 232^Th. The naturally present potassium can be present in food in the amounts of several dozen or several hundred Bq per one kilo of a consumed product [3]. It is known that gastrointestinal tract is one of the main ways in which radioisotopes enter human organism [4]; therefore, it is necessary to monitor the content of radioactive substances in various elements of environment, in particular those used for food production.

The analysis of radioactive isotopes content in food products and the assessment of annual consumption of these products provide the basis to estimate the size of radiation dose absorbed with food. On the basis of the size of absorbed doses, it is possible to assess potential health hazard to the people, who use food products from the analyzed area. In the event of exceeding or the risk of exceeding the permissible radiation doses, implementation of the appropriate corrective actions is necessary.

## Materials and methods

**Fig. 1:**
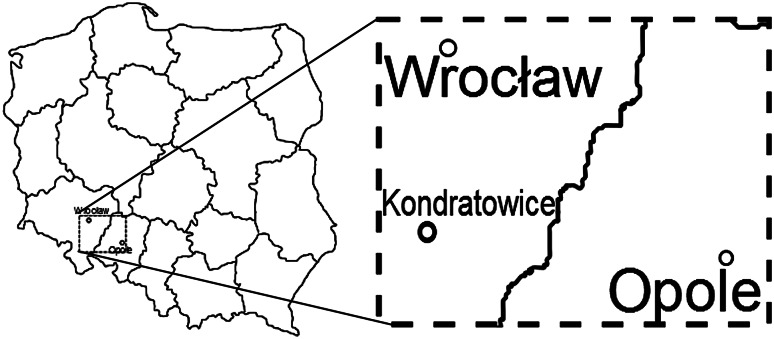
Location of experimental field of the seeding company KWS Lochów Polska Sp. z o.o. in Kondratowice

Activity concentration of gamma radionuclides was measured in edible parts of two varieties of potato, two varieties of corn and two varieties of sugar beet. The plants were collected from experimental field of the seeding company KWS Lochów Polska Sp. z o.o. in Kondratowice, 40 km from Wrocław (Dolnośląskie Province, Poland) (Fig. 1).

Two varieties of potato were used in studies: Inova and Bafana. Inova is a very early salad variety with early shaped tubers, long period of survival and storage. Medium-late Bafana variety with oval tubers is an ideal material for fast food industry. The analyzed corn variety was Ronaldinio, which in the period 2009–2011 was the most frequently cultivated one in Europe and the early grain variety Podium. The sugar beet variety is Primadonna, which has the highest sugar and root yield among the varieties registered in Poland in 2011, as well as Danuśka, which has the lowest content of harmful nitrogen in juice, among the varieties registered in 2011 [5].

Varieties of potatoes—Inova and Bafana were collected on 30 August 2012 while corn and sugar beet varieties were collected at 26 September 2012.

The collected plant samples were cleaned from soil, homogenized and dried at 105 °C to constant mass.

Also, soil samples from experimental fields area of 0.5 ha were taken into account. Samples of soil were taken with Egner’s test stick, in line with the methodology defined in PN-R-04031:1997 [6]. Samples from each field were mixed, homogenized and dried at 105 °C, prior to measurements taking.

The soils on sampling area were black soils.

The measurements of activity concentrations of ^ 40^K, ^137^Cs,^ 212^Pb,^ 214^Bi,^ 214^Pb,^ 226^Ra,^ 228^Ac,^ 231^Th and^ 235^U were carried out by means of a gamma-spectrometer with a germanium detector HPGe (Canberra) of high resolution: 1.29 keV (FWHM) at 662 keV and 1.70 keV (FWHM) at 1,332 keV. Relative efficiency: 21.7 %. Energy and efficiency calibration of the gamma spectrometer was performed with the standard solutions type MBSS 2 (Czech Metrological Institute, Prague, CZ) which covers an energy range from 59.54 to 1,836.06 keV. Geometry of calibration source was Marinelli (447.7 ± 4.5 cm^3^) with density 0.99 ± 0.01 g/cm^3^, containing^ 241^Am,^ 109^Cd,^ 139^Ce,^ 57^Co,^ 60^Co, ^ 137^Cs,^ 113^Sn,^ 85^Sr,^ 88^Y and^ 203^Hg. Geometry of samples container was Marinelli, 450 cm^3^. Measuring process and analysis of spectra were computer controlled with use of the software GENIE 2000.

## Results and discussion

The objective of the study was to define the value of the dose absorbed in consequence of consumption of popular food products for individual age groups. Also, translocation of ^ 40^K, ^137^Cs,^ 212^Pb,^ 214^Bi,^ 214^Pb,^ 226^Ra,^ 228^Ac,^ 231^Th and^ 235^U from soil to the tested plants was defined.

Translocation of radionuclides from soil to plants was described by transfer factors (TF) calculated on the basis of the formula 1 [7–9].

1 $$ {TF = \frac{{a_{p} }}{{a_{s} }}} $$

where *a*_p_ is the radionuclide activity concentration in the plant, and *a*_s_ is the activity concentration of the radionuclide in soil.

Table 1 contains characteristics of distribution of radionuclides specific activity concentration in the soil from experimental field. Maximum (Max), minimum (Min) values, lower quartiles (Q3), upper quartiles (Q1), mean values ($\overline{X}$), medians (Q2) and standard deviations (σ) are presented.

**Table 1:** Characteristics of distribution of radionuclides specific activity in the soil from experimental field

	^ 40^K	^ 137^Cs	^212^Pb	^214^Bi	^214^Pb	^226^Ra	^228^Ac	^231^Th	^235^U
Bq/kg d.m.									
$\overline{X}$	662	5.30	36.2	28.2	29.5	22.5	40.7	10.0	4.17
σ	38.2	0.25	3.15	1.80	2.71	13.9	2.92	1.96	1.07
Min	578	4.66	32.7	25.2	26.4	1.06	36.5	8.19	2.12
Max	731	5.64	44.6	31.4	35.7	47.5	48.6	14.7	5.58
Q_2_	660	5.33	35.2	28.5	28.7	22.8	40.4	9.39	4.30
Q_3_	643	5.25	34.2	27.0	27.5	15.3	39.0	8.74	3.43
Q_1_	686	5.48	36.8	29.1	31.4	29.2	41.8	10.2	5.14

**Fig. 2:**
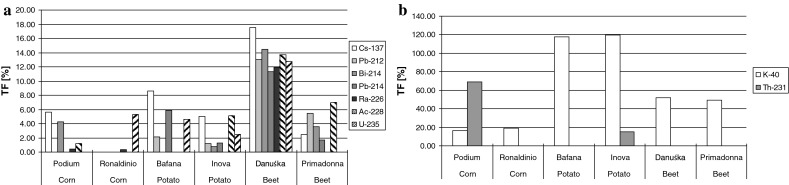
Transfer factor in the edible parts of the test plants expressed as a percentage for ^ 137^Cs,^ 212^Pb,^ 214^Bi,^ 214^Pb,^ 226^Ra,^ 228^Ac,^ 235^U radionuclides (graph **a**), transfer factor in the edible parts of the test plants expressed as a percentage for ^40^K,^ 231^Th (graph **b**)

The results of measurements of specific activity concentration of isotopes in soil were used in calculation of the transfer factors values (Fig. 2 a, b).

The calculated values of TF differed not only by the radionuclide type but also by varieties and species. The value of TF factor may depend on the type of a tested plant element [10]. The largest values were noted for natural isotopes ^ 40^K and^ 231^Th—in the first case amounting to 120 % of the isotope activity concentration in soil. Among the radioisotopes contained in edible parts of plants, anthropogenic ^ 137^Cs was identified, which content depends mainly on the activity concentration of this isotope in soil [11]. The values of transfer factor for this isotope oscillated between the values from below determination level for the Ronaldinio corn grains, to 17.5 % for Danuśka sugar beet.

The basis for calculations of the radiation dose absorbed in consequence of consumption of food products was the Council of Ministers Ordinance of 18 January 2005, regarding the limit doses of ionising radiation [12], defining the effective dose *E* [Sv/kg] expressed by the formula 2:

2 $$ {E = e(g) \cdot A}, $$

where e(*g*) is a unit effective dose for persons in a given age group [Sv/Bq], A is theactivity concentration of the radionuclide, which was absorbed with food [Bq/kg].

The e(*g*) values in conformity with the ordinance are presented in Table 2.

**Table 2:** Values of the committed effective doses of radionuclides in different age groups [12]

Nuclide	2–7 years old	7–12 years old	12–17 years old	>17 years old
Sv/Bq				
^40^K	2.10 × 10^−8^	1.30 × 10^−8^	7.60 × 10^−9^	6.20 × 10^−9^
^137^Cs	9.60 × 10^−9^	1.00 × 10^−8^	1.40 × 10^−8^	1.30 × 10^−8^
^212^Pb	3.30 × 10^−8^	2.00 × 10^−8^	1.30 × 10^−8^	6.00 × 10^−9^
^214^Bi	3.60 × 10^−10^	2.10 × 10^−10^	1.40 × 10^−10^	1.10 × 10^−10^
^214^Pb	5.20 × 10^−10^	3.10 × 10^−10^	2.00 × 10^−10^	1.40 × 10^−10^
^226^Ra	6.20 × 10^−7^	8.00 × 10^−7^	1.50 × 10^−7^	2.80 × 10^−7^
^228^Ac	1.40 × 10^−9^	8.70 × 10^−10^	5.30 × 10^−10^	4.30 × 10^−10^
^231^Th	1.20 × 10^−9^	7.40 × 10^−10^	4.20 × 10^−10^	3.40 × 10^−10^
^235^U	8.50 × 10^−8^	7.10 × 10^−8^	7.00 × 10^−8^	4.70 × 10^−8^

**Fig. 3:**
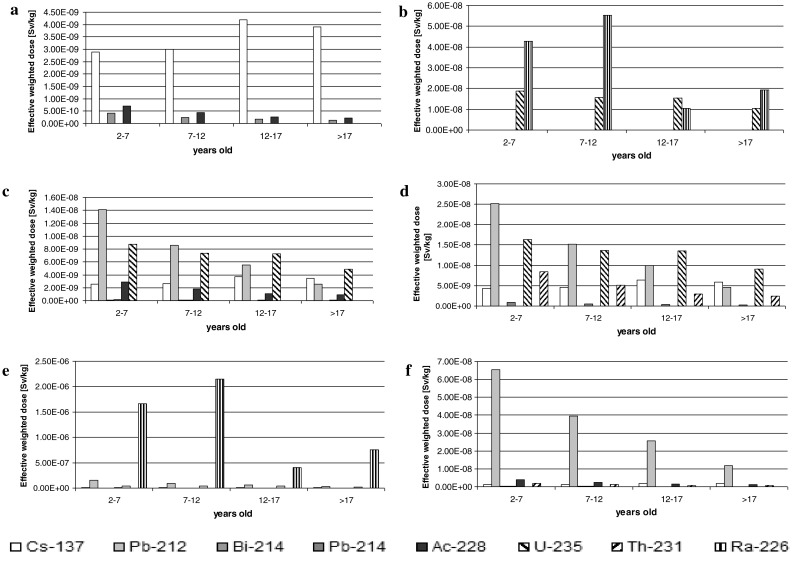
Effective weighted dose due to consumption of 1 kg of dry mass of a plant [Sv/kg], **a** Podium corn, **b** Ronaldinio corn, **c** Inova potato, **d** Bafana potato, **e** Sugar beet Danuśka, **f** Sugar beet Primadonna

The analysis of Fig. 3 shows that effective radiation doses for each isotope, caused by consumption of 1 kg of dry mass of a product, are at the level of micro- and sometimes nanosieverts. The highest radiation doses may affect children under 7 years old. For this group, the doses absorbed in consequence of consumption of 1 kg of food, converted into dry mass, were the highest for potatoes and sugar beet, up to hundredths of milisieverts. Values for other age groups oscillated within the ranges of 10^−3^–10^−4^ milisieverts (Fig. 3 a–f). Different doses were also noted between various plant elements; higher values were determined in storage organs of potatoes and beets and lower values in corn grains.

**Fig. 4:**
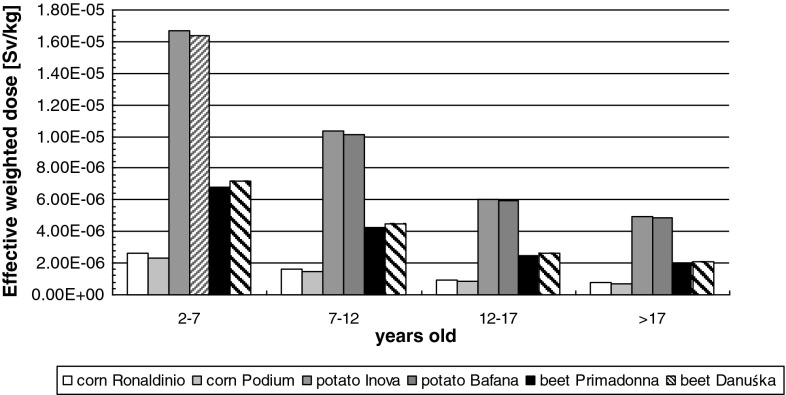
Effective weighted dose due to consumption of 1 kg of dry mass of a plant for different age groups for ^ 40^K radionuclide [Sv/kg]

High content of ^ 40^K in plants influences the value of the effective dose absorbed with the consumed food. The values related to the isotope are presented in Fig. 4.

The values presented in Table 2 were used to calculate the annual effective dose absorbed in consequence of the radionuclides present in food. For this purpose, the obtained results were calculated versus the annual consumption volume of a given product.

The annual consumption was calculated on the basis of data from Annual Statistical Book [13] by sharing annual consumption of a product by the current population of Poland, according to formula 3:

3 $$ {Sj = Sr/L \cdot 1 0 0 0}, $$

where *Sj* is the annual consumption kg/person, *Sr* is the annual consumption of product [tons], *L* is the current population in Poland.

The annual consumption of corn in Poland during the period 2010/2011 was 0.65 kg per person; however, the consumers’ preferences focused on potatoes, the consumption of which amounts to 111 kg, i.e. 27.1 kg of dry mass, assuming water content of 85.4 % [14]. In the case of sugar beets, the values were converted into sugar content, assuming that is makes 18.3 % of the mass (the values provided by KWS Lochów Polska Sp. z o.o.) and its annual consumption in 2011 was 39.7 kg per person [15]. The transfer of radionuclides from sugar beet to sugar was confirmed by measurement carried out for sugar samples available in stores. The results were similar to our results from calculation.

The annual effective dose was calculated by summing up the doses from the radionuclides, entering human organism during the period.

Table 3 presents values of total annual doses and percentage of ^ 137^Cs. It should be emphasizes that, together with the change of an age group, the percent share of ^137^Cs in the absorbed dose increases. This results from the constant conversion factor for this radionuclide. The values of other radionuclides decrease with the change of an age group.

**Table 3:** Values of total annual doses and percentage of ^137^Cs

	2–7 years old	7–12 years old	12–17 years old	>17 years old
Corn Podium				
^ 137^Cs [%]	0.12	0.20	0.49	0.55
Total dose [mSv]	0.0015	0.0010	0.0006	0.0005
Corn Ronaldinio				
^ 137^Cs[%]	BDL			
Total dose [mSv]	0.002	0.001	0.001	0.001
Potato Inova				
^ 137^Cs [%]	0.02	0.03	0.06	0.07
Total dose [mSv]	0.454	0.281	0.164	0.134
Potato Bafana				
^ 137^Cs [%]	0.03	0.04	0.11	0.12
Total dose [mSv]	0.447	0.277	0.162	0.132
Sugar beet Danuśka (sugar)				
^ 137^Cs [%]	0.10	0.14	0.42	0.41
Total dose [mSv]	0.259	0.193	0.089	0.084
Sugar beet Primadonna (sugar)				
^137^Cs[%]	0.02	0.03	0.07	0.08
Total dose [mSv]	0.197	0.122	0.071	0.058

During the analysis of the obtained results, assuming that an average person consumes different food products, an attempt was made to estimate the dose resulting from absorption of radionuclides from three popular products, i.e. Inova potato, Danuśka sugar beet and Ronaldinio corn grain. Having summed up total annual doses, we obtained the values of approximately 0.714 mSv/year for the age group 2–7 years old to 0.219 mSv/year for those over 17 years of age. The dose includes ^137^Cs (Table 4); however’ its content is much lower than other radionuclides.

**Table 4:** Absorption of radionuclides from three popular products, i.e. Inova potato, Danuśka sugar beet and Ronaldinio corn grain

	2–7 years old	7–12 years old	12–17 years old	>17 years old
mSv/year				
Total effective weighted dose	0.714	0.475	0.254	0.219
^ 137^Cs	0.0003	0.0003	0.0005	0.0004

This doses does not signify any threat to human health.

## Conclusions

On the basis of the obtained results and comparison of sources [16–18], one can state that in consequence of consumption of the selected food products, an average person may receive certain doses of both natural and artificial radioactive isotopes. However with point of view of existing norms, regardless of the age group, such doses do not pose threat to human health in consequence of consumption of the tested food products. ^137^Cs appeared among the determined radionuclides; however, its share in the absorbed dose is much lower than the doses from natural radioactive isotopes, in particular ^ 40^K, and it is on average 0.04 % of the limit effective dose, which for total population is 1 mSv/year [12].
